# Factors associated with age-related changes in oral diadochokinesis and masticatory function in healthy old adults

**DOI:** 10.1186/s12903-024-04214-9

**Published:** 2024-04-16

**Authors:** Se-Yeon Min, Nan-Sim Pang, Yu-Ri Kim, Sol-Ah Jeong, Bock-Young Jung

**Affiliations:** 1 Department of Dentistry, Graduate School, Yonsei University, Seoul, 03722 Korea; 2 Department of Advanced General Dentistry College of Dentistry, Yonsei University, 50 Yonseiro, Seodaemun-Gu, Seoul, 03722 South Korea

**Keywords:** Masticatory performance, Tongue-lip motor function, Swallowing function, Oral frailty

## Abstract

**Background:**

This cross–sectional study aimed to identify factors associated with age-related changes in masticatory performance (MP) and oral diadochokinesis (ODK) and to provide normal values in healthy old adults for the diagnosis of oral frailty.

**Methods:**

A total of 385 participants were divided into three age groups (Gr1-3): 20–64 years, 65–74 years, and ≥ 75 years. To investigate tongue-lip motor function, ODK was assessed as the number of repetitions of the monosyllables /pa/ta/ka/. Four questionnaires were used to assess subjective masticatory ability, cognitive ability, and psychological status. MP, bite force, and occlusal area were tested to assess dynamic objective masticatory function, and the number of remaining teeth and functional tooth pairs were determined to assess static objective masticatory function. Handgrip strength (HG), oral dryness, and tongue pressure (TP) were assessed to identify influencing factors. Intergroup differences were evaluated by ANOVA and the Kruskal‒Wallis test, and correlations between ODK and orofacial factors were evaluated.

**Results:**

This study revealed significant age-related declines in TP, HG, and ODK, especially after 65 years of age. Factors affecting MP were posterior teeth, the Eichner index, bite force, occluding area, the Korean Mini-Mental State Examination (KMMSE) score, and ODK. Each ODK syllable was associated with different factors, but common factors associated with ODK were MP, HG, and PHQ-9 score. For the syllables /pa/ta/, the Eichner Index, TP, and oral dryness were also associated. For the syllable /ka/ in Gr3, MP, TP, HG, oral dryness, and the KMMSE score were associated.

**Conclusions:**

These results could provide practical guidelines for oral rehabilitation in old adults and contribute to improving the understanding of age-related changes in oral function and the multidimensional nature of masticatory dynamics.

## Background

The age-related functional deterioration of orofacial structures, defined as oral frailty, has been a focus of research, and several masticatory parameters have been identified as important factors of masticatory performance (MP) in geriatrics [1–3]. A position paper of the Japanese Society of Gerodontology (JSG) classified the orofacial state into four stages, i.e., healthy, oral frailty, oral hypofunction, and oral dysfunction, and suggested that the stages of oral frailty and oral hypofunction could be restored to the previous stage by performing dental interventions before the irreversible conditions of the oral dysfunction stage [4]. Therefore, numerous previous studies have focused on establishing a clear, standardized diagnostic system and determining the interactive associations between oral and masticatory factors [3, 4].

Oral function has been investigated by assessing multiple factors, including oral hygiene, oral dryness, bite force (BF), tongue-lip motor function, tongue pressure (TP), swallowing function, the number of remaining teeth, the number of functional tooth pairs and MP, which can impair essential activities of daily living (ADLs) and contribute to physical frailty [2–6]. In addition, physical, psychological, neurologic, and structural factors are associated with “oral frailty”, and malnutrition is the most relevant factor in old adults [7, 8].

Swallowing disorders, or dysphagia, can be caused by aging, swallowing-related muscle degeneration, sarcopenia, physical frailty, and comorbidities, leading to malnutrition, aspiration pneumonia, significant mortality, and morbidity [9]. Swallowing requires the coordinated activity of many nerves and muscles of the mouth, lips, pharynx, and esophagus [10, 11]. In particular, oropharyngeal dysphagia encompassing the oral cavity, representing an initial voluntary component, is an essential domain [10].

The oral stage can be subdivided into two stages: the oral preparatory phase and the propulsive stage [10]. The preparatory phase is more complex for solid food than for liquid because the food should be transported over the teeth to be chewed and mixed with saliva and then positioned and swallowed similar to a liquid. At the initial stage of swallowing, the bolus is temporarily positioned within the anterior portion of the mouth, in front of the posterior wall of the transient cavity created by the middle portion of the tongue and the soft palate. Then, during the oral-propulsive stage, the bolus of fluid is positioned on the surface of the tongue, the tongue tip is in contact against the roof of the mouth, and the bolus is moved toward the pharynx through anterior-to-posterior flexion of the tongue [9]. Normal swallowing requires harmonized coordination among nerves and muscles, such as the superior laryngeal nerve, a branch of the vagus nerve, the tongue, and the suprahyoid muscle [9, 10].

Tongue-lip motor function, an indicator of orofacial function, is generally assessed using oral diadochokinesis (ODK) based on linguistic background [4, 11]. Decreased tongue-lip motor function can be a result of sensory impairments in brain function or perioral muscle function, such as Parkinson’s disease, traumatic injury, head and neck surgery, muscle atrophy, and malnutrition [4]. A few recent studies have reported that reduced ODK is associated with decreased swallowing function [12–15]. In the process of swallowing, the initial motion of the tongue is related to ODK /ta/, while the latter is similar to ODK /ka/ pronunciation; in particular, ODK /pa/ and /ka/ have been reported to be significantly related to swallowing function. The lips also help to create a tight seal that prevents food and liquids from leaking during the swallowing reflex and traps food in the buccal or labial sulci [10–12]. Moreover, some researchers have reported controversial outcomes regarding ODK parameters, e.g., that an ODK /pa/ score < 6.2 times/s is associated with swallowing problems, suggesting the importance of the maximum voluntary lip force [4, 11, 12]. There is neither sufficient evidence for the association of each component of ODK /pa/, /ta/, and /ka/ with changes in tongue-lip motor function, especially swallowing function, depending on aging, nor enough data to apply in clinical investigations. It is clear that tongue-lip motor function is crucial for swallowing and MP in the population [12, 15–17].

Decreased TP indicates a reduced ability to force the bolus toward the pharynx, suggesting impaired swallowing. The cutoff value for diagnosing decreased TP in the old population has been suggested to be 26–30 kPa [4, 8].

Several previous epidemiologic studies have reported that oral dryness, manifesting as a prodromal sign of oral frailty, is caused by various factors, such as medication, poor general health, aging and sex [4, 18]. However, there are controversial findings about the association between MP and salivary flow in vulnerable old individuals [19, 20]. A cross-sectional study on tongue-lip motor function and orofacial functions reported that xerostomia was negatively correlated with tongue-lip force and reduced ODK /ka/, increasing the likelihood of subjective swallowing difficulties in older individuals [11].

By establishing standard values of various masticatory functional factors, such as tongue-lip motor function, TP, and oral dryness, according to age, it will be possible to diagnose oral frailty or oral hypofunction quantitatively and qualitatively in the old population. However, there has been a lack of research on diagnostic reference values. The goal of this study was not only to assess normal data for various oral functional factors in old individuals with normal dentition but also to determine factors associated with age-related changes in MP and ODK. The null hypothesis of this study was that there are no significant differences in ODK or other oral functional factors among the age groups.

## Methods

### Study participants

Among the patients who visited the Department of Advanced General Dentistry of the Yonsei University Dental Hospital from August 2020 to January 2023, 385 participants (168 males and 217 females) who met the following criteria were included: (1) over 20 years old and capable of independent ADLs and (2) at least 20 remaining teeth, including fixed dental prostheses, without any chewing difficulties. Participants who (1) had a history of surgical intervention or radiation therapy in the head and neck area, (2) had ongoing dental treatment or removable dental prostheses, or (3) had diseases causing decreased oral function, such as stroke, dementia or temporomandibular disease, were excluded. Of the 420 participants, 35 individuals with insufficient recordings were also excluded. The sample size was determined using G*Power 3.1 software (Kiel University, Kiel, Germany) with an α of 0.05, a power of 0.8, and an effect size of 0.25. A total of 159 participants (*n* = 53/group) were required for data analysis. More data were collected even after securing the appropriate sample size considering the cross-sectional nature of the study. The participants were divided into 3 groups according to age: Group 1 (< 65 years; *n* = 184), Group 2 (65–74 years; *n* = 101), and Group 3 (≥ 75 years; *n* = 100).

### Study design

This cross-sectional study was conducted according to the guidelines of the Declaration of Helsinki and approved by the institutional review board committee of the Yonsei School of Dentistry (No. 2-2020-0047). For masticatory function evaluation, both subjective and objective assessments of various orofacial factors were performed. Methodologies for subjective and objective masticatory assessment identical to those used in our previous studies were applied [21, 22]. Two trained researchers conducted both objective and subjective masticatory examinations for all participants.

#### Objective assessment

Objective assessments can be categorized into static and dynamic domains. Static assessments included the number of remaining teeth, the Eichner Index of functional tooth pairs, MP, BF, HG, ODK, the occluding area (OA), and oral dryness.

### Tooth number, Eichner index, MP, TP, BF, HG, OA

The number of remaining teeth and the Eichner index of functional tooth pairs were determined by counting the teeth that were natural or restored, except for the third molars and root rests. MP was assessed by measuring the glucose concentration extracted from chewed gummy jelly (Gurucolum, GC, Tokyo, Japan) using a special device (Glucosensor GS-2, GC, Tokyo, Japan). TP was measured using a balloon-based device (TPM-01, JMS, Hiroshima, Japan). Participants were asked to press the balloon probe against the anterior hard palate, maintaining peak pressure for approximately 7 s, and the average of three trials was recorded. BF and OA were assessed utilizing a bite force analyzer (Dental Prescale 50 H, GC, Tokyo, Japan) [21]. HG, reflecting overall bodily strength, was tested using a handheld dynamometer (TKK 5401, Takei Scientific Instruments Co., Ltd., Tokyo, Japan) [22].

### ODK

ODK was assessed by recording the number of repetitions for the monosyllables /pa/, /ta/, and /ka/ per second to evaluate the movement capacity of the lips, the anterior region of the tongue and the posterior region of the tongue, respectively, using an automatic measuring device (Kenkou-kun Handy, Takei Scientific Instruments Co., Ltd., Tokyo, Japan). Each syllable was pronounced audibly for a total of 5 s, and the evaluation involved determining the number of syllables pronounced per second.

### Oral dryness

Oral moisture was measured at the center of the lingual mucosa approximately 10 mm from the tip of the tongue using an oral moisture-checking device (Mucus®, Life Co., Ltd., Saitama, Japan). The average value of three measurements was recorded. Oral moisture values range from 0 to 99.9, and values of ≥ 29.6, 28.0–29.5, and ≤ 27.9 are defined as normal, borderline dry mouth, and dry mouth, respectively [23].

#### Subjective assessments

Four questionnaires, the Food Intake Ability (FIA) questionnaire, Oral Health Impact Profile (OHIP)-14, Korean Mini-Mental State Examination (KMMSE), and Patient Health Questionnaire (PHQ)-9, were used to assess subjective masticatory ability, cognitive ability, and psychological status.

**FIA** was evaluated by asking the subjects to complete a questionnaire listing 14 kinds of food ranging from easy to difficult-to-chew food [22]. A five-point Likert scale was used to score masticatory ability as follows: 1 point, cannot chew at all; 2 points, difficult to chew; 3 points, cannot say either way; 4 points, can chew some; and 5 points, can chew well.

The **OHIP-14** is a commonly used questionnaire for assessing oral health-related quality of life (OHRQoL). This questionnaire evaluates seven items using a four-point Likert scale (0 = never, 1 = hardly ever, 2 = occasionally, 3 = fairly often, and 4 = very often). A lower total score indicates better OHRQoL [24].

The **KMMSE** consists of 30 questions. The score ranges from 0 to 30, and lower scores under 23 indicate significant cognitive impairment. The **PHQ-9** is used for assessing self-reported depression severity. PHQ-9 scores of 5, 10, 15, and 20 correspond to mild, moderate, moderately severe, and severe depression, respectively [25].

### Data analysis

All statistical analyses were carried out using SAS 9.4 (SAS Institute, Inc., Cary, NC) and R (version 4.0.0, R Foundation for Statistical Computing, Vienna, Austria) with a significance level of α = 0.05. Normality was assessed using the Shapiro-Wilk test. One-way analysis of variance (ANOVA) and the Kruskal‒Wallis test were used to compare normally distributed and nonnormally distributed continuous variables, respectively. The intragroup differences were analyzed by repeated-measures ANOVA with the Bonferroni post hoc correction. To determine the statistical correlation between ODK and masticatory function, multiple generalized linear analyses were performed. All of the methodologies were reviewed by an independent statistician.

## Results

### Demographic information

The participants consisted of 217 women (56.36%) and 168 men (43.64%), and the mean age was 61.30 years (Table 1).

**Table 1:** Demographic characteristics of participants

		Total	Gr1	Gr2	Gr3	p
subjects, N (%)		385 (100)	184 (47.79)	101 (26.23)	100 (25.97)	
Age (years)		61.30 ± 18.07	45.89 ± 13.39	70.01 ± 2.70	80.85 ± 4.83	
Sex (%) †	Male	168	81	46	41	0.8012
	Female	217	103	55	59	

†Chi-square test

### Subjective and objective masticatory function among age groups

Table 2 shows various significant differences regarding the tested orofacial factors. The Eichner index, TP, HG, and ODK /pa/ta/ka/ significantly decreased with increasing age. The number of remaining teeth, posterior teeth, OA, and BF differed significantly between Gr1 and Gr2 and between Gr1 and Gr3 but did not differ between Gr2 and Gr3. There was a significant decrease in FIA only between Gr1 and Gr2. Significant differences in the PHQ-9 score and oral dryness were observed between Gr1 and Gr3. The KMMSE score, MP, and OHIP-14 score presented no significant differences among the age groups.

**Table 2:** Assessment of variables of oral function among age groups

						Bonferroni correction		
		**Gr1**	**Gr2**	**Gr3**	**p** ††	**1 vs. 2**	**1 vs. 3**	**2 vs. 3**
Remaining teeth		27.60 ± 1.80	26.40 ± 2.60	26.00 ± 4.16	< 0.0001 *	0.0012 *	< 0.0001 *	0.0984
Posterior teeth		15.68 ± 1.78	14.92 ± 1.80	14.38 ± 2.08	< 0.0001 *	0.0054 *	< 0.0001 *	0.0522
Eichner index (A: B) †		180:4	95:6	88:12	0.0051 *			
MP		188.14 ± 65.93	186.07 ± 60.86	183.09 ± 54.04	0.2750	.	.	.
TP †††		36.29 ± 10.40	30.41 ± 8.45	25.39 ± 8.48	< 0.0001 *	< 0.0001 *	< 0.0001 *	0.0064 *
BF		855.83 ± 434.55	662.73 ± 358.16	671.58 ± 291.25	< 0.0001 *	0.0039 *	< 0.0001 *	1.0000
Occluding area		26.26 ± 14.05	20.74 ± 10.44	20.36 ± 8.47	0.0001 *	0.0045 *	0.0006 *	1.0000
HG		28.83 ± 9.49	25.78 ± 9.12	21.89 ± 7.89	< 0.0001 *	0.0363 *	< 0.0001 *	0.0009 *
ODK	/pa/	5.86 ± 0.83	5.17 ± 0.80	4.74 ± 0.83	< 0.0001 *	< 0.0001 *	< 0.0001 *	0.0042 *
	/ta/	5.95 ± 0.96	5.17 ± 0.80	4.57 ± 0.74	< 0.0001 *	< 0.0001 *	< 0.0001 *	< 0.0001 *
	/ka/	5.65 ± 0.81	4.93 ± 0.87	4.49 ± 0.84	< 0.0001 *	< 0.0001 *	< 0.0001 *	0.0030 *
Oral dryness		28.32 ± 1.66	28.59 ± 2.19	27.78 ± 3.88	0.0083 *	0.0.1887	0.0075 *	0.1.0000
FIA		67.24 ± 10.37	65.70 ± 9.18	62.71 ± 12.81	0.0217 *	0.0450 *	0.1062	1.0000
OHIP-14		5.31 ± 7.10	4.88 ± 6.93	4.76 ± 6.63	0.5258	.	.	.
KMMSE		27.87 ± 1.91	27.04 ± 3.09	25.58 ± 5.10	0.0914	.	.	.
PHQ-9		2.01 ± 2.97	2.87 ± 3.45	4.43 ± 5.50	0.0039 *	0.2865	0.0039 *	0.3258

*Notes* Gr = group; MP = masticatory performance; TP = tongue pressure; BF = bite force; HG = handgrip strength; ODK = oral diadochokinesis; FIA = food intake ability; OHIP-14 = Oral Health Impact Profile-14; KMMSE = Korean Mini-Mental State Examination; PHQ-9 = Patient Health Questionnaire-9

Mean ± SD

† Chi-square test

†† Kruskal‒Wallis-Wallis test

††† ANOVA

p * < 0.05

### Multiple linear regression analysis of associations with ODK

Table 3 presents multiple linear regression analysis results for factors associated with ODK. Significant associations were observed with the Eichner index, MP, TP, BF, HG, oral dryness, KMMSE score, and PHQ-9 score for both /pa/ and /ta/, and OA was also significant for /ta/. For /ka/, significant associations were present with MP, HG, and the PHQ-9 score.

**Table 3:** Multiple linear regression analysis of factors associated with ODK

	Dependent variable	Multiple model†			
		B	CI		p
/pa/	Remaining teeth	0.0211	-0.0326	0.0748	0.4394
	Posterior teeth	0.0207	-0.0392	0.0806	0.4969
	Eichner index	-0.6392	-1.0973	-0.1811	0.0065 *
	MP	0.0032	0.0014	0.0051	0.0007 *
	TP	0.0159	0.0028	0.0290	0.0177 *
	BF	0.0004	0.0000	0.0007	0.0299 *
	Occluding area	0.0096	-0.0002	0.0193	0.0545
	HG	0.0480	0.0250	0.0711	< 0.0001 *
	Oral dryness	0.0721	0.0105	0.1337	0.0219 *
	FIA	0.0066	-0.0032	0.0165	0.1863
	OHIP-14	-0.0032	-0.0197	0.0133	0.7016
	KMMSE	0.0409	0.0015	0.0803	0.0419 *
	PHQ-9	-0.3424	-0.6401	-0.0447	0.0243 *
/ta/	Remaining teeth	0.0224	-0.0311	0.0759	0.4108
	Posterior teeth	0.0284	-0.0313	0.088	0.3501
	Eichner index	-0.4944	-0.9593	-0.0296	0.0372 *
	MP	0.0035	0.0016	0.0054	0.0003 *
	TP	0.0137	0.0002	0.0272	0.0467 *
	BF	0.0005	0.0002	0.0009	0.0024 *
	Occluding area	0.0125	0.0025	0.0224	0.014 *
	HG	0.0521	0.0286	0.0756	< 0.0001 *
	Oral dryness	0.0655	0.0023	0.1287	0.0424 *
	FIA	0.007	-0.0031	0.0171	0.1751
	OHIP-14	-0.0154	-0.0321	0.0014	0.0728
	KMMSE	0.0464	0.01	0.0827	0.0128 *
	PHQ-9	-0.5519	-0.8517	-0.2522	0.0003 *
/ka/	Remaining teeth	0.0248	-0.0336	0.0831	0.4036
	Posterior teeth	0.0262	-0.0388	0.0913	0.4278
	Eichner index	-0.4113	-0.915	0.0925	0.1090
	MP	0.0021	0	0.0041	0.0492 *
	TP	0.0112	-0.0032	0.0256	0.1262
	BF	0.0003	-0.0001	0.0007	0.1172
	Occluding area	0.0066	-0.004	0.0173	0.2215
	HG	0.0623	0.0375	0.087	< 0.0001 *
	Oral dryness	0.0652	-0.0021	0.1325	0.0576
	FIA	0.0003	-0.0104	0.0111	0.9515
	OHIP-14	-0.0057	-0.0237	0.0122	0.5301
	KMMSE	0.0318	-0.0137	0.0774	0.1693
	PHQ-9	-0.4227	-0.7463	-0.0991	0.0107 *

*Notes* B = beta coefficient; CI = confidence interval; MP = masticatory performance; TP = tongue pressure; BF = bite force; HG = handgrip strength; ODK = oral diadochokinesis; FIA = food intake ability; OHIP-14 = Oral Health Impact Profile-14; KMMSE = Korean Mini-Mental State Examination; PHQ-9 = Patient Health Questionnaire-9

†Adjusted by sex, age, BMI

p * < 0.05

Table 4 shows the regression analysis results for ODK by age group. In Gr1, HG and the PHQ-9 score were significantly associated with ODK /pa/, while BF, OA, HG, and the PHQ-9 score were associated with ODK /ta/; BF, HG, and the PHQ-9 score were associated with ODK /ka/. In Gr2, MP was significantly associated with ODK /pa/, while MP and OA were significantly associated with /ta/, and no factors were associated with ODK /ka/. In Gr3, the Eichner index, MP, TP, HG, OHIP-14 score, and KMMSE score were significant factors for ODK /pa/, while the Eichner index, MP, TP, HG, OHIP-14 score, KMMSE score, and PHQ-9 score were significant factors for ODK /ta/; MP, TP, HG, oral dryness, and the KMMSE score showed significance for ODK /ka/.

**Table 4:** Multiple linear regression analysis of factors associated with ODK according to age

	Dependent variable	Gr1				Gr2				Gr3			
		B	CI		p	B	CI		p	B	CI		p
/pa/	Remaining teeth												
	Posterior teeth												
	Eichner index									-0.7666	-1.4339	-0.0992	0.0249 *
	MP					0.0033	0.0002	0.0063	0.0345 *	0.0052	0.0014	0.009	0.0079 *
	TP†									1.4090	0.3373	2.4807	0.0106 *
	BF												
	Occluding area												
	HG	0.047	0.0156	0.0785	0.0038 *					0.0723	0.0242	0.1204	0.0037 *
	Oral dryness												
	FIA												
	OHIP-14									-0.0382	-0.0757	-0.0008	0.0453 *
	KMMSE									0.0562	0.0090	0.1034	0.0202 *
	PHQ-9	-0.5732	-1.0954	-0.0511	0.0318 *								
/ta/	Remaining teeth												
	Posterior teeth												
	Eichner index									-0.6904	-1.3453	-0.0356	0.0391 *
	MP					0.0038	0.0008	0.0067	0.0140 *	0.0047	0.0011	0.0084	0.0118 *
	TP†									1.0289	-0.0174	2.0751	0.0538 *
	BF	0.0007	0.0002	0.0012	0.0052 *								
	Occluding area	0.0152	0.0020	0.0283	0.0244 *	0.0199	0.0001	0.0397	0.0491 *				
	HG	0.0489	0.0124	0.0854	0.0092 *					0.0738	0.0283	0.1194	0.0018 *
	Oral dryness												
	FIA												
	OHIP-14									-0.0568	-0.0911	-0.0225	0.0015 *
	KMMSE									0.0731	0.0292	0.1170	0.0014 *
	PHQ-9	-0.6774	-1.2784	-0.0765	0.0276 *					-0.508	-0.9806	-0.0354	0.0355 *
/ka/	Remaining teeth												
	Posterior teeth												
	Eichner index												
	MP									0.0050	0.0012	0.0088	0.0097 *
	TP†									1.3736	0.3068	2.4403	0.0123 *
	BF	0.0005	0.0001	0.0010	0.0160 *								
	Occluding area												
	HG	0.0485	0.0165	0.0805	0.0033 *					0.0974	0.0520	0.1428	< 0.0001 *
	Oral dryness									0.1152	0.0031	0.2274	0.0441 *
	FIA												
	OHIP-14												
	KMMSE									0.8596	0.3369	1.3824	0.0016 *
	PHQ-9	-0.5419	-1.0744	-0.0095	0.0461 *								

*Notes* Gr = group; B = beta coefficient; CI = confidence interval; MP = masticatory performance; TP = tongue pressure; BF = bite force; HG = handgrip strength; ODK = oral diadochokinesis; FIA = food intake ability; OHIP-14 = Oral Health Impact Profile-14; KMMSE = Korean Mini-Mental State Examination; PHQ-9 = Patient Health Questionnaire-9

*Adjusted by sex, age, BMI

†Log-transformed variable adjusted by sex, BMI

p * < 0.05

### Multiple linear regression analysis of associations with MP

Table 5 shows that several factors, including posterior teeth, the Eichner index, BF, OA, KMMSE score, and ODK /pa/ta/ka/, were significantly associated with MP. The regression analysis by age group indicated that different factors were associated with MP; BF and the PHQ-9 score for Gr1, TP, BF, OA, ODK /pa/ta/, and FIA for Gr2, and the Eichner index, BF, ODK /pa/ta/ka/, and KMMSE score for Gr3 (Table 6).

**Table 5:** Multiple linear regression analysis of factors associated with MP

Dependent variable	Multiple model†			
	B	CI		p*
Remaining teeth	2.5180	-0.5467	5.5828	0.1070
Posterior teeth	4.3815	0.9373	7.8257	0.0128 *
Eichner index	-47.7829	-77.0938	-18.4721	0.0015 *
TP	0.7139	-0.0069	1.4346	0.0522
BF	0.0454	0.0283	0.0625	< 0.0001 *
Occluding area	1.0641	0.5307	1.5976	0.0001 *
HG	0.9333	-0.3139	2.1804	0.1420
/pa/	13.7767	5.9157	21.6378	0.0007 *
/ta/	14.1787	6.5285	21.8289	0.0003 *
/ka/	7.3549	0.0269	14.6829	0.0492 *
Oral dryness	0.4872	-0.2839	1.2582	0.2145
FIA	0.1309	-0.5078	0.7695	0.6872
OHIP-14	-0.5130	-1.4114	0.3855	0.2623
KMMSE	3.3718	0.7648	5.9788	0.0116 *
PHQ-9	-6.7177	-26.4373	13.0018	0.5029

*Notes* B = beta coefficient; CI = confidence interval; MP = masticatory performance; TP = tongue pressure; BF = bite force; HG = handgrip strength; ODK = oral diadochokinesis; FIA = food intake ability; OHIP-14 = Oral Health Impact Profile-14; KMMSE = Korean Mini-Mental State Examination; PHQ-9 = Patient Health Questionnaire-9

†Adjusted by sex, age, BMI

p * < 0.05

## Discussion

While most studies in gerontology include individuals older than 60 or 65 years, age was set based on the general trend of classifying individuals as senior citizens at or above the age of 65 in this study. At the initial stage of this study, participants were divided into four age categories: Gr1 (20–44 years), Gr2 (45–64 years), Gr3 (65–74 years), and Gr4 (over 75 years). However, there were no significant differences in orofacial variables between Gr1 and Gr2. Therefore, the participants were divided into three groups: Gr1 (< 65 years old), Gr2 (65–74 years old) and Gr3 (≥ 75 years old). Given the cross-sectional nature of the study, careful attention was given to participant selection to avoid confounding effects on the validity and reliability of the research findings. The participants were healthy enough from the perspective of physical and mental conditions to manage ADLs and instrumental ADLs [26]. In addition, they had at least 20 teeth, which is considered a sufficient number of teeth to support masticatory function based on the short arch concept [4, 21, 26].

Muscle-related orofacial variables, such as TP, HG, and ODK, continuously decreased with age, while dentition-related variables, such as remaining teeth, functional tooth pairs, BF, and OA, showed significant changes after 65 years of age. In addition, the PHQ-9 score and oral dryness showed meaningful differences at 75 years of age. The continuous decrease in muscle-related orofacial factors is supported by previous studies showing that human age-related muscle atrophy begins at the age of 25 years and continuously progresses and accelerates after the age of 60 [11, 22, 27].

Despite significant differences in both dentition and muscle-related factors among age groups, there was no significant difference in MP or the OHIP-14 score. These findings can be interpreted as follows: MP can be recovered through functional adaptation to the oral environment, and the OHIP-14 score, a subjective masticatory factor, can also be maintained.

In this study, the ODK of three syllables, /pa/ta/ka/, differed significantly among the age groups, and the mean value of each syllable was lower than that in the JSG position paper [4], even though all participants were physically healthy with normal masticatory function (Table 2). Schimmel et al. observed that each age group represented different mean ODK values, which were lower than the cutoff values described in previous studies that did not consider the age of the population, the severity of frailty, or the dental status of the participants [11–13]. The need to re-evaluate the cutoff values based on ethnicity and language has been suggested for diagnosing ODK or oral frailty [11]. The findings of this study also suggest that age-related ODK thresholds should be redefined and customized based on mother tongue languages by integrating frailty or ethnicity.

Considering the association between ODK and orofacial functional factors, three common factors—MP, HG, and the PHQ-9 score—appeared to influence the ODK of /pa/ta/ka/. For the syllables /pa/ and /ta/, significant associations were observed for the Eichner index, BF, TP, and KMMSE score (Table 3). Several studies have reported controversial results that pronouncing the /pa/ syllable < 6.2 times/s is associated with swallowing difficulties [12] and that the /ka/ value is associated with subjective swallowing function [14] or with MP in denture wearers [17].

Why did the syllable /ka/ show different associated factors than the syllables /pa/ta/? The syllable /ka/ did not show a significant correlation with TP and showed a comparatively weak association with MP compared to the syllable /pa/ta/. Considering that TP measurements primarily focus on the anterior or middle dorsum of the tongue [12], the weak association between ODK /ka/ and TP can be supported by a previous study reporting that TP was associated with only ODK /ta/ [11]. In the transition from the oral to oropharyngeal phases, where the food bolus is transferred by the elevation and contraction of the tongue and soft palate, the involuntary sequential swallowing process is more related to the root of the tongue and the syllable /ka/ [10, 11, 14, 28, 29], which might explain the weaker associations between the syllable /ka/ and TP.

ODK /pa/ta/ was associated with the Eichner index and OA, which are related to well-functioning occlusion [10, 29–31]. Both /pa/ and /ta/ are classified as plosive sounds, but while /pa/ emphasizes the opening and closing of the lips, /ta/ involves more active tongue movement. The optimum position of the tongue is crucial to its functional effectiveness. Kotsiomiti et al. reported that abnormal positioning of the resting tongue is increasingly common as the number of natural teeth decreases [32]. Therefore, it can be assumed that dentition-related factors such as the Eichner index and OA area can potentially improve syllable pronunciation by stabilizing the structural tongue position.

The ODK values in Gr1 were associated with HG and the PHQ-9 score, but in Gr3, associations with TP, HG, MP and the KMMSE score appeared (Table 4). Notably, the PHQ-9 score, a psychological factor, affects ODK in individuals under 65 years of age, while the KMMSE score, an indicator of cognitive ability, affects individuals over 75 years of age. Although most of the participants in this study were psychologically and physically considered normal, the KMMSE was observed to influence ODK /pa/ta/ but not /ka/ (Table 3).

Lee et al. reported significant associations between MP and the KMMSE score in older individuals [21]; therefore, a significant multilateral association could be expected among the KMMSE score, MP and ODK. In reality, in Gr3, both the KMMSE score and ODK /ka/ emerged as significant factors associated with MP, and ODK /pa/ showed a more significant association than in Gr2, where ODK /pa/ta/ was distinctively associated with MP (Table 6). This means that the muscles related to ODK /ka/ become weaker after weakening of the voluntary muscles related to /pa/ta/, which could be serial events in the old population [17, 33]. In Gr1, the PHQ-9 score was significantly associated with MP and ODK. This result is in line with a study showing that subjective criteria, such as psychological condition, are significant factors of MP in younger individuals [22, 34].

Posterior teeth, OA, the Eichner index, BF, ODK, and the KMMSE score exhibited significant associations with MP (Table 5). The Eichner index, indicating the locations and numbers of functional tooth pairs, showed a significant association with MP regardless of age, which is supported by previous studies [3, 35]. However, in the intragroup analyses, it showed no significance except for in Gr3 (Table 6). This result suggests that the Eichner index becomes more pronounced as a determinant of MP due to the significantly fewer remaining teeth of those in Gr3 (Tables 2 and 6).

**Table 6:** Multiple linear regression analysis of factors associated with MP according to age

Dependent variable	Gr1				Gr2				Gr3			
	B	CI		p	B	CI		p	B	CI		p
Remaining teeth												
Posterior teeth												
Eichner index									-42.5013	-78.9876	-6.0150	0.0229 *
TP					1.8973	0.2065	3.5880	0.0283 *				
BF	0.0238	0.0011	0.0465	0.0397 *	0.0742	0.0416	0.1069	< 0.0001 *	0.0523	0.0090	0.0957	0.0185*
Occluding area					2.5139	1.3615	3.6662	< 0.0001 *				
HG												
/pa/					19.2554	1.4430	37.0677	0.0345 *	16.5585	4.4724	28.6445	0.0079 *
/ta/					22.4188	4.6846	40.1531	0.0140 *	16.3964	3.7346	29.0582	0.0118 *
/ka/									16.2255	4.0337	28.4173	0.0097 *
Oral dryness												
FIA					1.5369	0.0751	2.9988	0.0395 *				
OHIP-14												
KMMSE									3.0643	0.3933	5.7352	0.0251 *
PHQ-9	-47.2094	-81.2865	-13.1322	0.0071 *								

*Notes* Gr = group; B = beta coefficient; CI = confidence interval; MP = masticatory performance; TP = tongue pressure; BF = bite force; HG = handgrip strength; ODK = oral diadochokinesis; FIA = food intake ability; OHIP-14 = Oral Health Impact Profile-14; KMMSE = Korean Mini-Mental State Examination; PHQ-9 = Patient Health Questionnaire-9

*Adjusted by sex, age, BMI

p * < 0.05

TP consistently decreased across age groups, and no direct correlation between TP and MP was found. There seem to be controversial results regarding the association between MP and TP, indicating a positive association [3, 17]or no association [22]. This discrepancy in the association between TP and MP might be due to heterogeneity in study design, including the selection of comparison groups, latency, and ascertainment of diagnosis.

Oral dryness could be a risk factor for reduced swallowing function and MP. Some studies have shown that the stimulated salivary flow rate is positively associated with MP [19, 36], while other studies have reported that excessive saliva may not improve MP but that sufficient saliva is mandatory for chewing and swallowing [3, 6]. This study did not show any association between MP and oral dryness because most participants in this study were relatively healthy.

A few limitations of this study should be addressed. First, one of the original purposes of this study was to evaluate swallowing ability for the early identification of dysphagia or swallowing difficulties using ODK [11, 12, 15, 24, 37]. However, due to the complexity of swallowing mechanisms, an actual swallowing assessment was not performed. Therefore, more practical testing methods, such as the Repetitive Saliva Swallowing Test (RSST) and Eating Assessment Tool (EAT)-10, may be necessary for further research [11, 13, 14].

Second, despite this study having characteristics of a cross-sectional study, participants were selected from a single institution based on strict criteria to avoid confounding effects on the validity and reliability of the research findings, which may have introduced bias. Further studies based on multicenter trials with definitive inclusion criteria should be designed for more representative data and precise analysis. Additionally, various modifications of the subject criteria and population size depending on the investigated factors are needed to reveal the mutual causal relationships among various influencing factors.

## Conclusions

Under the limited conditions of this study, the mean values of the tested orofacial parameters showed significant differences depending on the age group. The ODK /pa/ta/ka/ values were significantly correlated with MP regardless of age group. For individuals over 75 years of age, the Eichner index, indicating the number of functional tooth pairs, and cognitive ability appeared to be significant factors influencing ODK and MP.

## Data Availability

The datasets used and/or analyzed during the current study are available from the corresponding author upon reasonable request.

## Abbreviations

ODK: oral diadochokinesis
TP: tongue pressure
MP: masticatory performance
BF: bite force
OA: occluding area
HG: hand grip
KMMSE: Korean Mini-Mental State Examination
OHIP-14: Oral Health Impact Profile-14
PHQ-9: Patient Health Questionnaire-9
ADLs: Activities of daily life
